# Supplementary material for: Determination of Modified QuEChERS Method for Chlorothalonil Analysis in Agricultural Products Using Gas Chromatography–Mass Spectrometry (GC-MS/MS)
Source: Foods. 2023 Oct 16;12(20):3793. doi: 10.3390/foods12203793 (PMC10606406; doi:10.3390/foods12203793)
Supplement: Supplementary file 1 [file foods-12-03793-s001.zip › foods-2606177-supplementary.pdf]

foods

MDPI

Supplementary Material

Determination of Modified QuEChERS Method for Chlorothalonil Analysis in Agricultural Products using GC-MS/MS

Da-Young Yun [1], Ji-Yeon Bae [1], Chan-Woong Park [2], Gui-Hyun Jang [1] and Won-Jo Choe [1,*]

[1]Food Safety Evaluation, Pesticide and Veterinary Drug Residues Division, National Institute of Food and Drug Safety Evaluation, Ministry of Food and Drug Safety, Cheongju 28159, Korea; dyyun96@korea.kr (D.-Y.Y.); jiyeon0962@korea.kr (J.-Y.B.); arion@korea.kr (G.-H.J.)

[2]Center for Food and Drug Analysis, Busan Regional Office of Food and Drug Safety, Busan 47537, Korea; pcw0324@korea.kr

*Correspondence: aragaya06@korea.kr; Tel.: +82-43-719-4206

Table S1. Analytical conditions for the GC-MS/MS of chlorothalonil

**Instrument**			
GC	7890B GC system (Agilent Technologies, Santa Clara, CA, USA)		
MS/MS	GC/MS Triple Quad (Agilent Technologies, Santa Clara, CA, USA)		
**GC conditions**			
Column	DB-5MS (30 m×0.25 mm, 0.25 μm)		
Flow rate	1.5 mL/min (He 99%)		
Injection volume	1 μL		
Injection mode	splitless		
Oven temp.	Rate (°C/min)	Temperature (°C)	Hold (min)
	Initial	90	1
	30	180	4
	20	300	3
**MS/MS condition**			
Ionization mode	Electron ionization (EI)		
Transfer line temp.	280℃		
Ion source temp.	280℃		

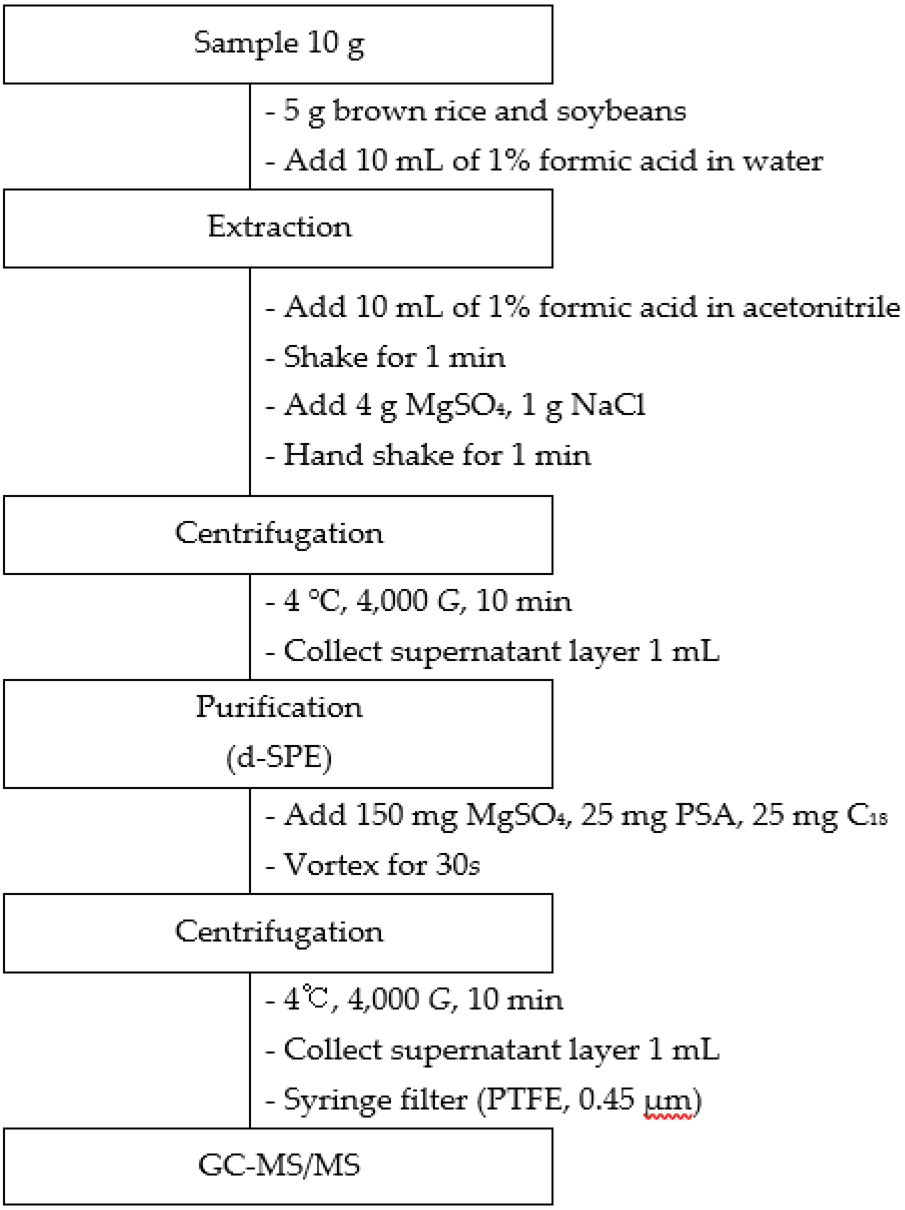

Figure S1. Experimental flow for chlorothalonil analysis in agricultural products.

Table S2. Comparison of the extraction data of different solvents and three QuEChERS salts in soybean for chlorothalonil, Solution A (water and acetonitrile), Solution B (1% formic acid in water and 1% formic acid in acetonitrile), and Solution C (1% acetic acid in water and 1% acetic acid in acetonitrile)

	Recovery ± CV[a] (%)		
	Original	AOAC 2007.01	EN 15662
Solution A	20.7±10.1	15.6±3.7	N.D.
Solution B	109.7±7.8	65.2±4.4	65.9±5.5
Solution C	73.3±4.3	40.0±9.3	71.0±7.8

[a] Average coefficient of variation
